# Nitrogen starvation causes lipid remodeling in *Rhodotorula toruloides*

**DOI:** 10.1186/s12934-024-02414-0

**Published:** 2024-05-17

**Authors:** Shekhar Mishra, Anshu Deewan, Huimin Zhao, Christopher V. Rao

**Affiliations:** 1 Department of Chemical and Biomolecular Engineering, DOE Center for Advanced Bioenergy and Bioproducts Innovation, Carl R. Woese Institute for Genomic Biology, University of Illinois Urbana-Champaign, Urbana, IL USA; 2 Departments of Chemistry, Biochemistry, and Bioengineering, University of Illinois Urbana-Champaign, Urbana, IL 61801 USA

**Keywords:** Oleaginous yeast, Lipid accumulation, Transcriptomics, Lipidomics

## Abstract

**Background:**

The oleaginous yeast *Rhodotorula toruloides* is a promising chassis organism for the biomanufacturing of value-added bioproducts. It can accumulate lipids at a high fraction of biomass. However, metabolic engineering efforts in this organism have progressed at a slower pace than those in more extensively studied yeasts. Few studies have investigated the lipid accumulation phenotype exhibited by *R. toruloides* under nitrogen limitation conditions. Consequently, there have been only a few studies exploiting the lipid metabolism for higher product titers.

**Results:**

We performed a multi-omic investigation of the lipid accumulation phenotype under nitrogen limitation. Specifically, we performed comparative transcriptomic and lipidomic analysis of the oleaginous yeast under nitrogen-sufficient and nitrogen deficient conditions. Clustering analysis of transcriptomic data was used to identify the growth phase where nitrogen-deficient cultures diverged from the baseline conditions. Independently, lipidomic data was used to identify that lipid fractions shifted from mostly phospholipids to mostly storage lipids under the nitrogen-deficient phenotype. Through an integrative lens of transcriptomic and lipidomic analysis, we discovered that *R. toruloides* undergoes lipid remodeling during nitrogen limitation, wherein the pool of phospholipids gets remodeled to mostly storage lipids. We identify specific mRNAs and pathways that are strongly correlated with an increase in lipid levels, thus identifying putative targets for engineering greater lipid accumulation in *R. toruloides*. One surprising pathway identified was related to inositol phosphate metabolism, suggesting further inquiry into its role in lipid accumulation.

**Conclusions:**

Integrative analysis identified the specific biosynthetic pathways that are differentially regulated during lipid remodeling. This insight into the mechanisms of lipid accumulation can lead to the success of future metabolic engineering strategies for overproduction of oleochemicals.

**Supplementary Information:**

The online version contains supplementary material available at 10.1186/s12934-024-02414-0.

## Background

The unicellular microorganism yeast is a promising platform for industrial-scale fermentations of value-added compounds. As a model yeast, *Saccharomyces cerevisiae* has been extensively characterized and engineered to produce a wide variety of bioproducts [1, 2]. However, *S. cerevisiae* is not an ideal host for overproducing lipids and lipid-related chemicals, also called oleochemicals. Oleaginous yeasts such as *Yarrowia lipolytica* and *Rhodotorula toruloides*, which can accumulate a much higher fraction of their biomass as lipids, are theoretically better platforms for producing oleochemicals [3, 4]. The basal metabolism of these yeasts results in a higher yield of lipids per substrate consumed, which is required to make such industrial fermentations economically viable. The red yeast *R. toruloides* is capable of accumulating as high as 70% of its biomass as lipids, while also allowing high cell density cultures, thus resulting in high titers of lipid production [5, 6]. Besides a high flux in lipid biosynthesis, *R. toruloides* is also known to show high tolerance towards inhibitory compounds, specifically those found in lignocellulosic biomass hydrolysate [7–9]. This tolerance combined with an ability to consume hexose and pentose sugars, both found in lignocellulosic biomass hydrolysate, suggests that *R. toruloides* can serve as a better host for converting lignocellulosic sugars into value-added compounds.

As *R. toruloides* accumulates high levels of lipids and carotenoids, both of which are synthesized from acetyl-CoA, *R. toruloides* has the potential to serve as a platform strain for producing a variety of compounds synthesized from acetyl-CoA. Previous studies have explored overproducing lipids in *R. toruloides* through a variety of strategies like inhibiting fatty acid degradation, increasing the flux of acetyl-CoA or other biosynthesis reactions in lipid pathways [6, 10]. Other work has focused on the production of fatty alcohols and triacetic acid lactone, both of which are synthesized from acetyl-CoA [11, 12]. The strategies implemented in most metabolic engineering studies so far focus on similar strategies of reducing lipid catabolism, increasing precursor supply, and introducing a heterologous enzyme (push–pull-block strategy).

While *R. toruloides* appears to be a promising host for the biomanufacturing of oleochemicals, the microorganism poses several challenges. It is considered a non-model yeast and as such, its metabolism is not well understood. While the accumulation of lipids in low nitrogen conditions has been well reported, the molecular basis of this phenotype has not been clearly elucidated [13]. Thus, to harness the full potential of *R. toruloides* in metabolic engineering applications, it is desirable to understand the mechanism of lipid accumulation.

Within systems biology, transcriptomic analysis is a robust analytical technique providing insights into the transcriptional machinery of an organism. Other studies endeavoring to find the molecular basis of lipid accumulation in *R. toruloides* have also employed transcriptomic analysis for their investigations. Coradetti et al. performed transcriptomic analysis of a library of *R. toruloides* loss-of-function mutants to identify putative genes affecting lipid metabolism in the yeast [14]. Zhu et al. employed a multi-omic method where genomic, transcriptomic and proteomic data were integrated to identify correlations between lipid accumulation and nitrogen compound recycling [13]. Recently, Jagtap et al. performed a transcriptomic and metabolomic analysis of *R. toruloides* grown on different sugars, identifying regulation patterns in central metabolic pathways as a result of growth on different substrates [15]. In other yeasts such as *Y. lipolytica,* multi-omic analysis, which included a genome-scale model, metabolite profiling, lipidomic analysis and transcriptomic data from RNA-Seq, identified the origin of lipid accumulation in flux rewiring within the amino acid metabolism [16]. However, in *R. toruloides*, no such integrated transcriptomic and lipidomic analysis has been performed so far.

In this study, we undertake a multi-omic investigation of lipid accumulation in *R. toruloides*. Nitrogen-sufficient and nitrogen-deficient conditions were studied to contrast a neutral phenotype against a lipid accumulation phenotype. The cells grown in both conditions were harvested for both transcriptomic and lipidomic analyses. As a primary analysis, clustering analysis of the transcriptomic data was qualitatively correlated against a similar trend in lipidomic data. Hierarchical clustering of the lipid data, however, showed a different clustering pattern than that seen from RNA-Seq clustering. Multi-omic analysis of the transcriptomic and lipidomic data indicated a lipid remodeling event wherein glycerophospholipids pathways are repressed whereas most of the carbon is shifted towards storage lipids. Our work represents the first comprehensive study integrating transcriptomic and lipidomic analyses of lipid accumulation phenotypes in *R. toruloides*.

## Results

### Experimental design for studying the lipid accumulation phenotype

**Fig. 1:**
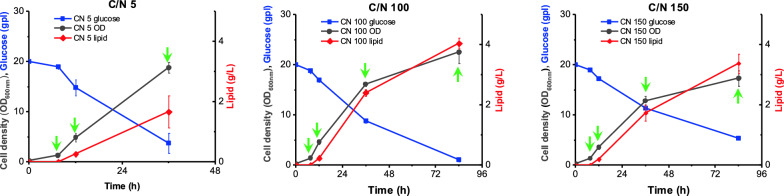
Experimental design scheme depicting the growth curves of *R. toruloides* IFO0880 in media with different C/N ratios. The green arrows in each plot represent the times when culture samples were collected for transcriptomic and lipidomic analysis

The growth and lipid accumulation phenotypes of *R. toruloides* IFO0880 were studied in three different media conditions. The three conditions were formulated to contain C/N ratios of 5, 100 and 150. A C/N ratio of 5 represented a baseline medium with nitrogen in sufficient concentration; a similar value was chosen to grow seed cultures in a previous study [17]. A study from 2020 tested the lipid accumulation capabilities of *R. toruloides* grown in media formulations ranging between C/N of 60 to 120 [5]. In the current study, a C/N ratio of 100 was chosen as a nitrogen-deficient media formulation known to promote lipid accumulation. Additionally, media with a C/N ratio of 150 was also included to study the effects of extreme nitrogen starvation. Samples were collected at three time points during growth: 8 h (early exponential phase), 12 h (mid-exponential phase) and 36 h (early saturation phase). For the two nitrogen-deficient conditions (C/N of 100 and 150), an additional sample at 88 h of growth was added (Fig. 1).

### Transcriptomic analysis of lipid accumulation dynamics

**Fig. 2:**
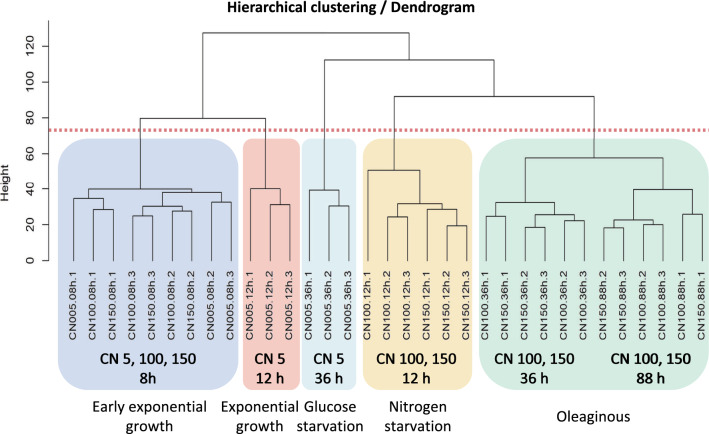
Hierarchical clustering performed on the time-based RNA-Seq analysis of *R. toruloides* cultures. The samples are shaded using different color boxes to lump together samples from different phases of growth. From left to right: Early exponential, Exponential, Glucose starvation, Nitrogen starvation and Oleaginous growth phases

**Fig. 3:**
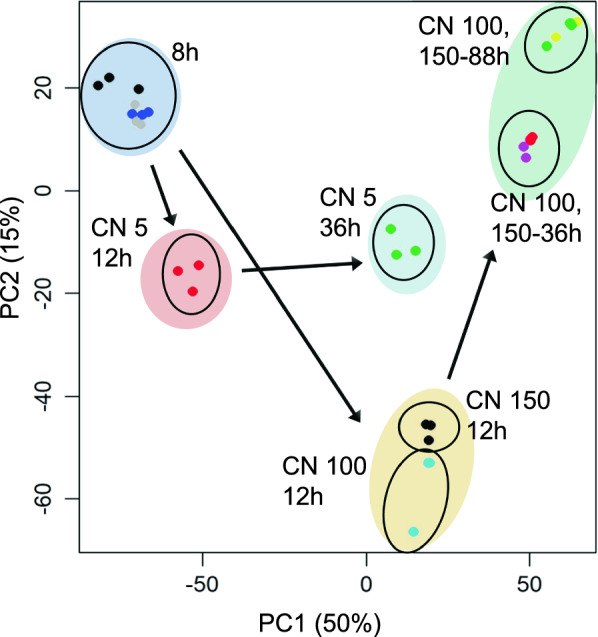
Principal component analysis of the RNA-Seq dataset showing the clustering of nitrogen-sufficient and nitrogen-deficient cultures. Additionally, cultures also cluster together based on the growth phase, with a clear divergence emerging at the exponential growth phase cultures (12 h of growth)

RNA-Seq analysis was used to inspect the differential gene expression in *R. toruloides* IFO0880 across different conditions and time points as described above. Hierarchical clustering of the RNA-Seq data showed that during the early exponential growth phase, when nitrogen is still available in all three media conditions, the transcriptomes of cells grown in the different media conditions cluster together (Fig. 2). As nitrogen depletion took effect during mid-exponential phase, the cultures grown in C/N of 100 and 150 began to diverge from C/N of 5, where nitrogen was still expected to be available. This divergence is highlighted even further in the early and late-saturation samples where nitrogen-depleted conditions cluster together. Principal component analysis (PCA) aided in improved visualization and analysis of this divergence where the lipid-accumulating and non-lipid-accumulating samples clearly follow different trajectories and cluster separately (Fig. 3). A clustered heatmap of the RNA-seq data was plotted for gene expression data collected across different media conditions and different time points (Additional File 1: Figure S1). The mRNAs were clustered into three groups – either showing a decrease in expression when switching from high nitrogen to low nitrogen, approximately constant expression during the switch, or showing an increase in expression when switching from high nitrogen to low nitrogen. The cluster identities are located in Additional File 2: Table S1 under the column RNA-seq Cluster ID.

**Fig. 4:**
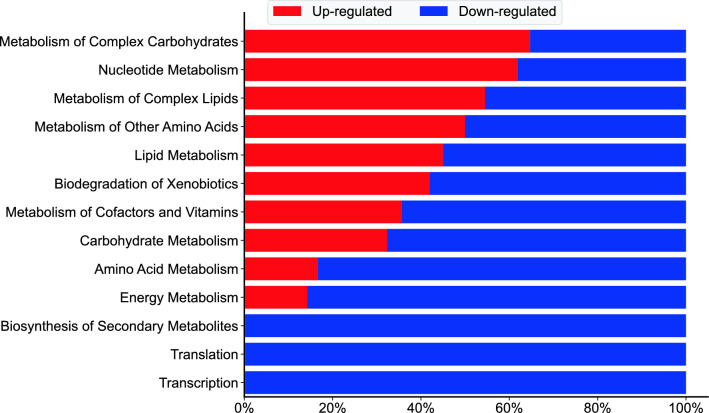
Gene set analysis of nitrogen limitation in *R. toruloides* IFO0880. The differentially expressed genes were sampled at a timepoint of 36 h and contrasted between nutrient limited conditions of C/N 100 and 150 versus C/N 5 (which served as a baseline). Since the same set of genes were differentially expressed in both C/N 100 and 150 in comparison with C/N 5, the conditions could be plotted interchangeably

RNA-Seq data were used to identify the subset of genes that were differentially expressed in conditions of nutrient starvation compared to baseline cultures. A threshold of greater than twofold change in gene expression (which includes up- and down-regulation) was employed. From the identified differentially expressed genes, the fraction of up- and down-regulated genes in all annotated pathway classes for *R. toruloides* IFO0880 were calculated and displayed as a horizontal bar graph. Figure 4 shows one such analysis for the comparison of differentially expressed genes between C/N of 100 and 150 compared to C/N of 5 at the 36-h time point. Since the same set of genes were differentially expressed in both C/N 100 and 150 in comparison with C/N 5, the conditions could be plotted interchangeably. The figure shows the metabolic classes with the highest fraction of up-regulated genes at the top and those with the highest fraction of down-regulated genes at the bottom. Inspection of this plot shows that during nitrogen starvation in C/N 100 and 150, the metabolic processes of translation, transcription and secondary metabolism were entirely down-regulated. Genes involved with amino acid metabolism also see a major fraction of differential expression as down-regulation. This trend has also been noticed in *Y. lipolytica* where nitrogen starvation significantly down-regulated amino acid metabolism [16]. However, in contrast with the findings of the *Y. lipolytica* study, genes involved in lipid metabolism were more differentially regulated in *R. toruloides* compared to the baseline condition. The study in *Y. lipolytica* reported minimal regulatory impact of nitrogen starvation on lipid metabolic genes, whereas our data indicated significant regulation in *R. toruloides*. The gene set analysis for differentially expressed genes at the 36-h time point was also contrasted to similar analysis for differentially expressed genes at the 8-h and 12-h time points (Additional File 3: Figure S2). As expected from the PCA plot in Fig. 3, at the 8-h mark, both media conditions showed very similar expression profiles and very few differentially expressed genes or pathways (Figure S2-A). A greater number of differentially expressed pathways began to emerge at the 12-h mark between strains grown in C/N 100 (or 150) compared to C/N 5, with the metabolism of complex carbohydrates, complex lipids and secondary metabolites showing significant upregulation (Figure S2-B). While secondary metabolism appeared to be completely down-regulated at the 36-h time point (Fig. 4), that of complex lipids and carbohydrates continued to be up-regulated under nitrogen deficient conditions, suggesting an important role in lipid accumulation.

### Lipidomic analysis of lipid accumulation dynamics

Liquid chromatography–mass spectrometry (LC–MS) was used to perform lipidomic analysis of extracted lipids from *R. toruloides* IFO0880 cells. The cells were harvested at the time points denoted by green arrows in Fig. 1. This allowed a time-based inspection of the lipidome for both lipid-accumulating and non-lipid-accumulating phenotypes.

**Fig. 5:**
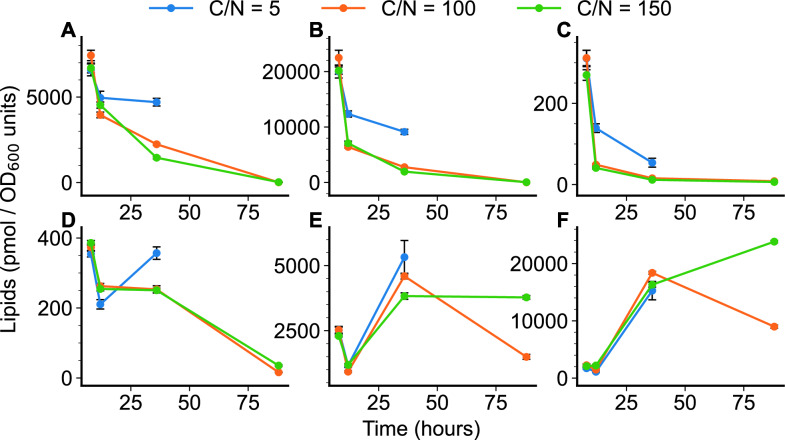
Lipidomic analysis of *R. toruloides* IFO0880 strain cultivated in different growth media containing C/N ratios of 5, 100 and 150, and sampled at various timepoints. Shown here are quantified lipid classes of all three growth conditions sampled at 8 h, 12 h, 36 h and 88 h of growth. A) Phosphatidylcholine (PC), B) Phosphatidylethanolamine (PE), C) Phosphatidylglycerol (PG), D) Phosphatidylinositol (PI), E) Diacylglycerol (DAG), and F) Triacylglycerol (TAG)

At the earlier time points, the measured lipid concentrations followed a similar trend to the expected clustering behavior observed from RNA-Seq data shown in Fig. 2 and Fig. 3. Lipid concentrations for the early exponential samples (8h growth) showed similar levels for all major lipid classes (Fig. 5). The divergence of lipid profiles at the exponential (12h sample) and saturation phase (36h sample) was noticeable in the phospholipid pools (Fig. 5A-D). Most glycerophospholipids (phosphatidylcholine (PC), phosphatidylethanolamine (PE), phosphatidylinositol (PI) and phosphatidylglycerol (PG)) were higher in C/N of 5 compared to C/N of 100 or 150 (Fig. 5 A-D, Additional File 4: Figure S3).

At the late saturation phase of oleaginous lipid accumulation, samples were only collected for C/N of 100 and 150, and, hence, lipid measurements were only available for lipid accumulating phenotypes. At this late stage of growth, the fraction of lipids within the cells is mostly TAG. Analysis of TAG levels within the two lipid-accumulating phenotypes shows a divergence in TAG levels with C/N 150 continuing to store carbon as TAG even after 88 h of growth, while TAG levels show a decrease in C/N 100 at the same time point (Fig. 5F, Additional File 5: Figure S4). Extracellular measurements (Fig. 1) show that the strain cultivated in C/N 100 media has consumed most of the glucose by this point in growth while significant amounts of glucose remain unconsumed in C/N 150. This measurement combined with the lipidomic dataset suggests that in the C/N 150 media, *R. toruloides* continues to accumulate carbon into TAG much longer, while the strain grown in C/N 100 media, having depleted most of its sugar substrate, potentially relies on lipid remodeling to generate carbon for continued survival. Within the set of annotated mRNA transcripts that were differentially expressed, the glycerol dehydrogenase was up-regulated in the C/N 100 condition at 88 h compared to the C/N 150 sample, suggesting increased activity in glycerolipid catabolism.

**Fig. 6:**
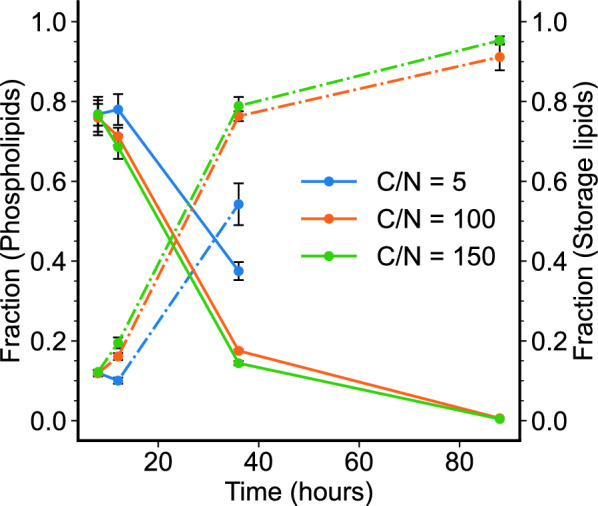
Variation in the fraction of total lipid pool in *R. toruloides* IFO0880 at each time point and for each growth condition. Two fractions are calculated: fraction of phospholipids (solid line) and fraction of storage lipids (dash-dot line)

Further inspection of the distribution of lipids showed that in all three growth conditions, the distribution of lipids shifts from mostly phospholipids towards mostly storage lipids. At the start of each growth culture (before nitrogen starvation takes effect), phospholipids dominate the fraction of intracellular lipids. As each growth condition progresses with time, the fraction of phospholipids was seen to subside and replaced almost entirely by storage lipids (Fig. 6). While culture conditions of C/N 100 and 150 closely resembled each other in trajectory, even C/N 5 showed a similar trend, albeit with different values.

A shift in the total intracellular lipids from phospholipids to storage lipids further reinforces the notion of an active lipid remodeling process as nitrogen starvation starts to take effect. Studies in the model oleaginous yeast, *Y. lipolytica*, have demonstrated that nitrogen limitation causes an overall increase in lipid pools in that organism [16, 18]. They show that nitrogen limitation in *Y. lipolytica* causes little change to transcriptional regulation in lipid metabolism and instead modulates amino acid metabolism. The overall increase in lipid levels has been attributed to an overflow of carbon into lipid biosynthetic pathways [16]. In contrast, however, the results shown in Fig. 4 and Fig. 6 suggest that nitrogen starvation in *R. toruloides* is accompanied by significant lipid regulation.

**Fig. 7:**
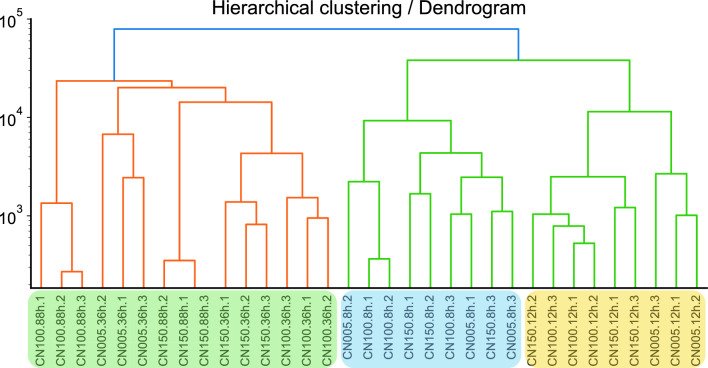
Hierarchical clustering performed on the time-based lipidomic analysis of *R. toruloides* cultures

A hierarchical clustering analysis of the lipidomic data was performed (Fig. 7), which shows that the 88-h and 36-h time points cluster similarly while the 8-h and 12-h time points cluster together. The latter suggests that the divergence in the RNA-Seq data that appears at 12 h of growth manifests in lipid phenotypes with a time delay, which is generally observed in RNA-metabolite trends [19].

### Multi-omic integrated analysis of lipid accumulation

So far, all literature reported investigations on the lipid accumulation phenotype of *R. toruloides* IFO0880 were performed using individual omic analyses, namely transcriptomic and lipidomic analysis in isolation. However, each of these datasets only present a partial picture of the biological processes governing the oleaginous yeast’s behavior. The transcriptomic dataset indicates snapshots of the gene expression profile of the cells, whereas the lipidomic dataset conveys a snapshot of the lipidome. Thus, a systematic method of analysis that reasonably integrates both datasets was sought for analysis.

**Fig. 8:**
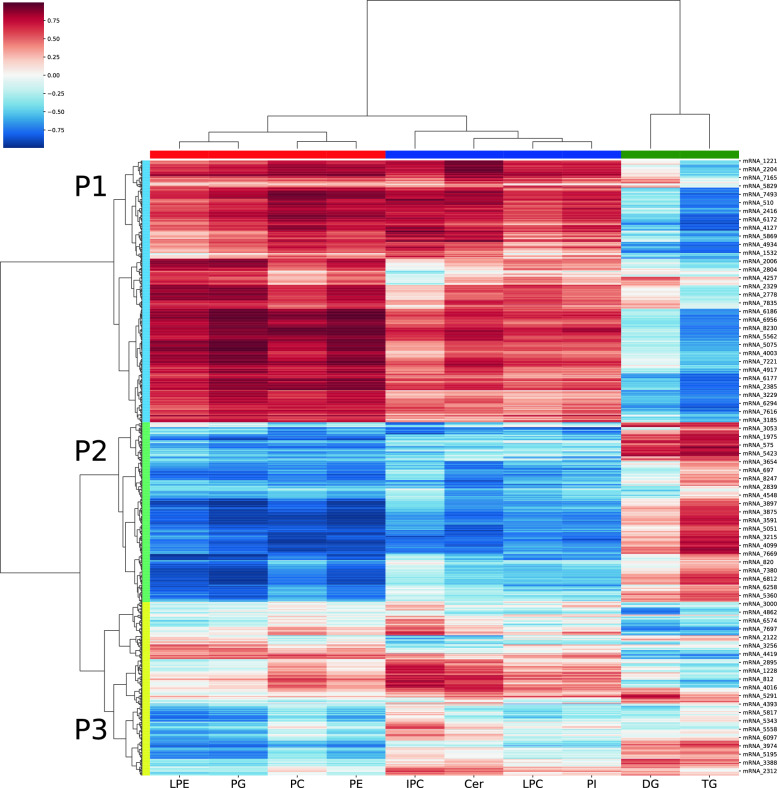
Cluster map capturing the correlations between measured transcripts and lipids, constructed for each condition with the horizontal axis representing the mRNA transcripts and the vertical axis consisting of the lipids. It shows the correlations between lipids concentrations and mRNA transcript levels across all media conditions and time points. The cluster identities can be found in Table S1 under the column Multi-omics Cluster ID

An integrative method of analysis previously described in Hsu and coworkers [20] was employed wherein transcriptomic and metabolomic profiles were analyzed together in the form of a cluster map. The 4 quadrants of such a cluster map were then imposed onto a pathway diagram to convey the correlations between nodes (metabolites) and edges (reactions) of the graph (metabolic pathway). We adopted a similar approach to analyze our data and visualize the correlated nodes and edges with the goal of formulating better hypotheses about the lipid accumulation pathway. A cluster map capturing the correlations between measured transcripts and lipids was constructed for each condition with the horizontal axis representing the mRNA transcripts and the vertical axis consisting of the lipids. The cluster map (Fig. 8) shows the correlations between lipids concentrations and mRNA transcript levels across all media conditions and time points. The cluster identities can be found in Table S1 under the column Multi-omics Cluster ID.

**Fig. 9:**
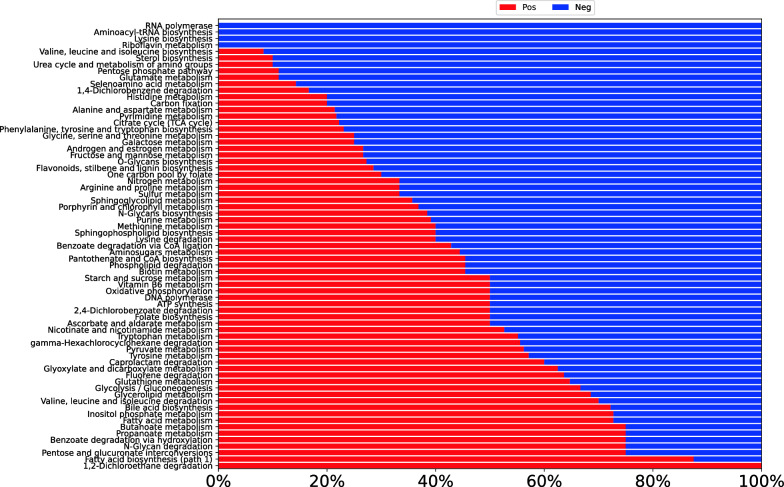
Pathway enrichment analysis. The list of mRNAs associated with a known reaction were fractioned into groups that are either negatively or positively correlated with TAG levels

The cluster map (Fig. 8) demonstrated that lipids and mRNAs both cluster into 3 groups each. The two mRNA clusters demonstrating either mostly negative or mostly positive correlations with TAGs (cluster P1 in the top-right and cluster P2 in the center-right in Fig. 8, respectively) were of particular interest. To explore the pathways that these mRNAs were associated with, a pathway enrichment analysis (similar in method to the gene enrichment analysis shown in Fig. 4) was conducted. The list of mRNAs associated with a known reaction were fractioned into groups that are either negatively or positively correlated with TAG levels (Fig. 9). The pathways related to RNA polymerase and aminoacyl-tRNA biosynthesis were fully negatively correlated with TAG levels, in agreement with the earlier observation of complete down-regulation of transcription and translation at the 12-h and 36-h time points (Figure S2 and Fig. 4). Pathways with greater than 50% positive correlation with TAG levels included the expected pathways in glycerolipid metabolism, fatty acid metabolism and fatty acid biosynthesis. Surprisingly, the pathway associated with inositol phosphate metabolism showed strong positive correlation with TAG despite the decrease in phosphatidylinositol (PI) levels during lipid accumulation (Fig. 5). Phosphatidylinositols are known to be associated with signaling roles and well-studied in yeasts such as *S. cerevisiae*. They specifically have been identified to influence the metabolism of storage lipids [21]. Analyzing the overlap between cluster identities of each mRNA from the clustermap analysis of just RNA-Seq data (Figure S1) compared to the clustermap analysis of the multi-omic data (Fig. 8) showed an interesting trend (Additional File 6: Figure S5). The mRNA cluster positively correlated with TAG levels (cluster P2 in Fig. 8) was inhabited by only mRNA that increased in expression when switching from high-nitrogen to low-nitrogen state. Similarly, the mRNA cluster negatively correlated with TAG levels (cluster P1 in Fig. 8) was mostly inhabited by mRNAs that decreased in expression when switching from high-nitrogen to low-nitrogen.

## Discussion

The non-model yeast *R. toruloides* is a promising chassis for metabolic engineering due to its ability to accumulate lipids at a much higher fraction than the model yeasts [22]. However, the lipid accumulation phenotype is generally induced under nutrient starvation conditions, where growth is compromised, leading to a tradeoff between lipid levels and biomass [5, 23]. Identifying the mechanism that governs accumulation is thus crucial for any effort to engineer a platform strain that can be employed for overproduction of oleochemicals [13]. In this work, we performed a comprehensive study of lipid accumulation phenotypes in *R. toruloides* by integrating transcriptomic and lipidomic analyses to identify the metabolic and regulatory processes that contribute the most to lipid accumulation for engineering purposes. We observed that the transcriptomic profiles for nitrogen-rich and nitrogen-deficient cultures clustered separately, with the divergence of profiles emerging as early as the exponential growth phase. The lipidomic profiles, however, showed greater similarity between the two types of growth conditions. Lipidome trends measured in the C/N of 5 trailed those of C/N 100 or 150 with a time-delay, presumably mirroring the trend of nitrogen consumption. Pathway visualization techniques demonstrated a map of increased regulation within the lipid biosynthesis pathway wherein neighboring reactions were either up- or down-regulated during lipid accumulation. These localized regulations along with trends observed within the lipidomic data proved that the oleaginous yeast *R. toruloides* displayed lipid remodeling during the accumulation phase instead of a uniform increase in all lipids.

Nitrogen starvation was applied in two different carbon-to-nitrogen ratios (C/N of 100 and 150) but both cultures showed similar transcriptomic responses compared to the baseline condition of C/N of 5. Clustering analysis of transcriptomic data showed a clear divergence of trajectories between the baseline and the nitrogen-starved conditions, with the split occurring from the 12-h timepoint itself (Fig. 3). While the divergence of transcriptomic profiles grew more prominent at the saturation phase timepoint of 36 h, the intracellular lipid profiles showed much less distance in the clustering patterns (Fig. 7, Additional File 1: Figure S1 and Additional File 2: Figure S2). This similarity of lipidomes as the cultures progressed in time can be explained by the fact that, over time, each growth condition (including C/N of 5) would become more nitrogen deficient because nutrients get used up by the cells with nothing being replenished. Further, the intracellular phospholipid profiles of C/N 5 culture at the 36-h mark resemble the profiles of the C/N 100 and 150 at the earlier 12-h measurements, suggesting that the C/N 5 condition experiences a similar form of nitrogen starvation at 36 h of growth as that faced by C/N 100 and 150 at about 12 h of growth.

The lipidomic timeseries profiles for all 3 conditions followed similar trends (Fig. 5) with a drop in mostly phospholipid-based lipidome being accompanied by a rise in mostly storage lipid-based one (DAG and TAG). This trend was demonstrated in Fig. 6 where the fraction of phospholipids dropped with increase culture time for all 3 growth conditions, while the fraction of storage lipids increased. Thus, unlike a trend of overall increase in lipids seen in *Y. lipolytica* [16], the increase in lipid pools in *R. toruloides* from nitrogen starvation resulted mainly in the increase of storage lipids. The selective decrease/increase of different lipid pools during the lipid accumulation phase suggested that lipid accumulation is accompanied by strong regulation of the lipid biosynthetic pathways. This contrasts with the findings in *Y. lipoytica* where regulation of the lipid pathways exerted very weak control over the lipid accumulation phenotype [16].

To identify the lipid biosynthetic pathways, whose up- or down-regulation most significantly influenced this observed lipid remodeling, the two ‘omic datasets were analyzed in an integrated manner. A cluster map enabled the binning of lipids and lipid biosynthetic reactions that showed a similar increase or decrease (Fig. 8). Pathway visualization (Figure S6) showed a clear shutdown of phospholipid biosynthesis pathways under lipid accumulation conditions, which agreed with the decreasing phospholipid levels observed in lipidomic data. More interestingly, the pathway maps also showed that lipid accumulation was accompanied with a positive correlation in the acylglycerol formation from the dihydroxyacetone phosphate (DHAP) pathway, indicating that the increased carbon into the TAG and DAG pools originated from the acylation of the glycerol-3-phosphate backbone.

The multi-omic integrative analysis enabled the identification of reactions that were regulated in a coordinated manner with an increase in storage lipids. The trend of the increase in lipids was observed in the direction of only storage lipids rather than a net increase of all intracellular lipid pools. Future studies can leverage the knowledge of these regulated pathways for developing engineering strategies. The strategy of multi-omic analyses for identifying lipid accumulation phenotypes has been employed in other organisms [24]. A study by Ajjawi et al. identified a putative set of transcriptional factors (TFs) that governed lipid production in the microalga *Nannochloropsis gaditana* under nitrogen-deficient conditions [25]. CRISPR-based knockdown of this putative set helped identify one TF that resulted in higher lipid accumulation without affecting growth. Such a method could be adapted to *R. toruloides* using CRISPR-interference systems with the goal of discovering global transcriptional factors that result in a lipid overproducing platform strain.

## Conclusions

In this study, we performed a multi-omic analysis of the lipid accumulation phenotype in the oleaginous yeast *R. toruloides* IFO0880 under nitrogen starvation. Transcriptomic analysis indicated divergent timeseries profiles of the baseline growth media (C/N of 5) compared to the nitrogen-deficient media (C/N of 100 and 150). Lipidomic analysis showed dissimilar lipid profiles at the start with the C/N 5 lipidome eventually converging towards the C/N 100, 150 lipidomes as nitrogen levels decreased over time. Multi-omic analysis of the growth conditions suggested that nitrogen starvation causes the lipidome to remodel with most of the glycerophospholipid pool shifting towards storage lipids. Thus, in batch culture conditions in *R. toruloides*, we observed a selective increase in storage lipids during nitrogen starvation instead of an overall increase of all lipids.

## Methods

### Strains, media, and growth construction

*R. toruloides* IFO0880, mating type A2, was obtained from the NITE Biological Resource Center in Japan (NBRC 0880). YPD medium (10 g/L yeast extract, 20 g/L peptone, and 20 g/L glucose) was used for growth of *R. toruloides* precultures. A single colony from a YPD agar plate was inoculated into 2 mL of YPD liquid medium to obtain *R. toruloides* seed cultures. Seed cultures were then used to inoculate 25 mL of media with a defined carbon-to-nitrogen ratio in a 125-mL baffled shake flask with a starting OD_600_ of 1. The optical density at 600 nm or OD_600_ was used to monitor the cell density in liquid cultures. OD_600_ of 1.0 corresponds to roughly 10^7^ cells per mL. The cells were then grown at 30 °C and 250 rpm. The formulation of media for different C/N ratios is listed in Additional File 8: Table S2.

### Sample extraction for RNA-Seq

Seed cultures at exponential phase were collected and centrifuged at 6000 × g for 3 min at 4 °C. Supernatant was discarded and the pellets were resuspended in 1 mL of ddH_2_O. Seed cultures then used to inoculate 25 mL of growth media with defined carbon–nitrogen ratios in a 125-mL baffled shake flask with a starting OD_600_ of 1 and incubated at 30 °C and 250 rpm. Growth experiments are performed with three biological replicates. Samples were collected at defined timepoints as described in Sect. 3.1. The cell cultures containing a total OD of 30 were collected in centrifuge tubes and centrifuged at 6000 × g for 3 min at 4 °C. Supernatant was discarded and pellet was used for RNA extraction. Total RNA was extracted using the RNeasy mini kit (Qiagen, Hilden, Germany) as previously described, with a slight modification [26, 27]. The *R. toruloides* cell pellet was resuspended in 350 µL of Buffer RLT from the RNeasy mini kit (Qiagen, Hilden, Germany). Approximately 500 µL of acid-washed glass beads (acid washed, 425–600 μm; Sigma, St. Louis, MO, USA) was added and homogenized using a FastPrep-24 homogenizer (MP Biomedicals, Irvine, CA, USA), beaten at a speed of 5 m/s for 30 s six times with cooling on ice between beatings. The cell lysates were purified according to the kit’s protocol titled “purification of total RNA from yeast.” Extracted RNA was then treated with Turbo RNase-free DNase kit (ThermoFisher, Waltham, MA, USA) according to the manual and purified again with the RNeasy mini kit protocol titled “RNA clean up.” The stranded RNAseq libraries were prepared with Illumina’s TruSeq Stranded mRNA Sample Prep kit. The libraries were quantitated by qPCR and sequenced on one lane for 101 cycles from one end of the fragments on a HiSeq 4000 (Illumina, San Diego, CA, USA). Fastq files with 100 bp reads were generated and demultiplexed with the bcl2fastq v2.17.1.14 Conversion Software (Illumina, San Diego, CA, USA).

### RNA-Seq data analysis

To obtain gene expression profiles during growth of *R. toruloides* IFO0880 on different substrates, total RNA was extracted, and a mRNA focused library was sequenced. Adaptor sequences and low-quality reads were trimmed using Trimmomatic [28]. Trimmed reads were analyzed for quality scores using FastQC [29]. Reads were mapped to the *R. toruloides* IFO0880 v4.0 reference genome (NCBI Accession GCA_000988875.2) with STAR version 2.5.4a [14, 30]. Between 95 and 98% of the reads were successfully mapped to the genome for each sample. Read counts were calculated using featureCounts from the Subread package, v1.5.2 [31]. Differential expression analysis was performed on the reads counts in R v4.0.5 (Cite R core team) using edgeR v3.32.1 and limma v3.46.0 [32, 33]. The iDEP software suite was also used for preliminary analysis of gene expression data [34]. Graphical representation of expression data was constructed using R packages: PCAtools v2.2.0, gplots v3.1.1, and Glimma v2.0.0 [35–37]. Before plotting heatmaps, the data was normalized row-wise (using the scale function in R), first by centering (subtracting the row mean from each value) and then scaling (dividing each data point by row’s standard deviation). Heatmaps were plotted using heatmap.2 function from gplots. Genome sequence, gene models, and functional annotation of *R. toruloides* was downloaded from the DOE Joint Genome Institute’s Mycocosm portal [14, 38]. The raw fastq RNA-seq files have been deposited at https://www.ncbi.nlm.nih.gov/bioproject/PRJNA1000066 (Bioproject ID: PRJNA1000066).

### Sample preparation for lipidomics analysis

At the time of sampling, cell concentration of the culture was measured as OD_600_/mL. One mL of cell culture was harvested, washed once with 150 mM ammonium bicarbonate (ABC) buffer (pH = 8), then resuspended in 500 µL ABC, and lysed open with approximately 200 µL of 0.5 µm zirconium glass beads via high-speed vortexing for 30 min. For lipid extraction, 2 OD_600_ units of cell lysate were added to a glass tube containing 200 µL ABC, 1 mL of 2:1 chloroform:methanol and 12 µL of an internal standard cocktail (mole amounts of each lipid in the cocktail listed in Additional File 9: Table S3). The glass tube was vortexed in a Fisherbrand MultiTube Vortexer (Thermo Fisher Scientific, Waltham, MA) for 2 h at 2500 rpm. After the phases were clearly separated, the chloroform layer was separated into a fresh vial and dried overnight. The dried lipid extract was resuspended in 100 µL of 4:2:1 isopropanol:methanol:chloroform. 10 µL of the lipid extract was injected on an LC–MS instrument (Vanquish UHPLC and Q-Exactive Orbitrap, Thermo Fisher Scientific, Waltham, MA).

### Lipidomic data generation and data analysis

The LC–MS protocol was followed as described in [39]. LC separation was performed on a Thermo Accucore Vanquish C18 + column (2.1 × 150 mm, 1.5 µm) with mobile phase A (60% acetonitrile: 40% H2O with 10 mM ammonium formate and 0.1% formic acid) and mobile phase B (90% isopropanol: 10% acetonitrile with 10 mM ammonium formate and 0.1% formic acid) and a flow rate of 0.25 mL/min. The linear gradient was as follows: 0 min, 60% A; 12 – 13.5 min, 0% A; 14 – 16 min, 60% A. The gas flow rates and MS1/MS2 scan parameters were followed exactly as listed in [39]. Data processing of the.RAW files generated from LC–MS runs was performed using the MS-DIAL software [40]. Identity of lipids was ascertained by comparing spectra to an in-house database of lipid molecules. Finally, quantification was performed using a one-point calibration where the peak intensity of each lipid molecule was normalized to the intensity of the representative lipid for its class within the spiked-in internal standard cocktail. This normalized value was multiplied to the absolute mole amount of the internal standard. The lipidomic data after processing and quantification is available in the units pmol/OD_600_ in Additional File 10: Table S4.

### Multi-omic data analysis

The multi-omic data analysis and visualization were performed using the seaborn and matplotlib libraries in Python [41, 42]. The clustermaps and pathway enrichment analysis plots were visualized using in-house scripts that employed the above two libraries. For pathway visualization, Escher maps were used that were developed in a previous study describing a genome-scale model in *R. toruloides* [43, 44].

### Supplementary Information

**Additional file 1. Figure S1.** Clustered heatmap of the RNA-seq expression data collected across different media conditions and different timepoints. The x-axis represents all the samples with individual replicates denoting the different growth media conditions and timepoints of sampling. The hierarchical dendrogram for the x-axis was computed using Euclidean distances between each pairwise combination of the samples’ mRNA expression profile. The y-axis denotes the subset of mRNA that had accompanying annotations. The hierarchical dendrogram for the y-axis was computed using the Pearson’s correlation matrix computed between pairwise combination of each mRNA (which was expressed as a numeric vector containing its expression count in each sample). The mRNA and samples were each assigned cluster identities (C1, C2, C3 and S1, S2, S3, S4, S5 respectively) as well as color labels along the left and top edges to distinguish the different clusters more easily. Further, a label of “High-N” and “Low-N” was assigned to cluster samples that were estimated to be in the nitrogen-sufficient or nitrogen-deficient regimes, respectively. From inspection of data, the mRNA belonging to cluster C1 showed a decrease in expression when switching from high nitrogen to low nitrogen media, cluster C2 showed an approximately constant expression across high and low nitrogen conditions, and cluster C3 showed an increase in expression when switching from high nitrogen to nitrogen starvation conditions. The information of mRNA identities that belong in these three clusters can be found in Additional File 2: Table S1.

**Additional file 2.** **Table S1.** Clustering identities of the listed mRNA.

**Additional file 3. Figure S2.** Gene set analysis of nitrogen limitation in R. toruloides IFO0880. The differentially expressed genes were sampled at a timepoint of A). 8 hours, and B). 12 hours, and contrasted between nutrient limited conditions of C/N 100 and 150 versus C/N 5 (which served as a baseline). Since the same set of genes were differentially expressed in both C/N 100 and 150 in comparison with C/N 5, the conditions could be plotted interchangeably

**Additional file 4. Figure S3.** Lipidomic analysis of IFO0880 strain cultivated in different growth media containing C/N ratios of 5, 100 and 150, and sampled at various timepoints. Quantified lipid classes of all three growth conditions sampled after A) 8 hours, B) 12 hours and C) 36 hours of growth.

**Additional file 5.** **Figure S4.** Lipidomic analysis of IFO0880 grown in C/N 100 and 150 culture conditions and sampled at a timepoint of 88 hours (oleaginous phase).

**Additional file 6.** **File S5.** Overlap of cluster identities of mRNA. The x-axis displays the 3 major mRNA clusters in the clustermap integrating multiomics data. The y-axis displays the distribution of these mRNA into the 3 major mRNA clusters from the RNA-seq clustering analysis.

**Additional file 7. File S6.** The reactions and metabolites (lipids) found in each of these quadrants were then assigned a color and these colors were superimposed onto a pathway map depicting the lipid biosynthetic pathways, extracted from a genome-scale model of R. toruloides (Dinh et al). The pathway visualization shown here is for C/N of 150. The Escher maps for visualization were adapted from the genome-scale model developed by Dinh et al. Reactions displayed using green are correlated positively with storage lipid increase (DAGs and TAGs), whereas those displayed with red are negatively correlated with storage lipid accumulation. The linear chain of reactions upstream of storage lipid synthesis were mostly positively correlated as expected. The reactions of phospholipid synthesis that utilize the same precursors as storage lipids, thus withdrawing flux from storage lipids synthesis are negatively correlated with storage lipid increase. This observation was verified from the lipidomic data where phospholipid values dropped in the oleaginous phase where DAG and TAG increased. A pattern of selective regulation of reactions within lipid biosynthetic pathways in the context of storage lipid accumulation was observed. The phospholipid biosynthetic pathways were negatively correlated to storage lipid accumulation in both maps (reaction pathway on the right), whereas reactions upstream of the storage lipid synthesis for acylation of the glycerol backbone were upregulated in both (bottom left). These patterns supported the observation that lipid accumulation during nitrogen starvation was accompanied by a rerouting of carbon flux towards storage lipids and away from glycerophospholipids. G3PD1i_c, glycerol-3-phosphate dehydrogenase (NAD); DHAPt_c_rm, dihydroxyacetone phosphate transport; DHAPAT_rm, Dihydroxyacetone phosphate acyltransferase; AGNPR_rm, Acylglycerone-phosphate reductase; GLYC3Pt_c_rm, glycerol 3-phosphate transport; G3PAT_rm, Glycerol-3-phosphate acyltransferase; AGPAT_rm, 1-Acyl-sn-glycerol-3-phosphate acyltransferase; PAP_rm, PA phosphatase; DGAT_rm, Diacylglycerol acyltransferase; CDPDAGS_rm, CDP-diacylglycerol synthase; PSSA_rm, PS synthase; PAILS_rm, PI synthase; INOSTt_c_rm, myo-inositol transport; MI1PP_c, myo-inositol 1-phosphatase; M1PS_c, myo-inositol-1-phosphate synthase; CHOLPT_rm, Cholinephosphotransferase; ETHAPT_rm, Ethanolaminephosphotransferase; PEMT_rm, PE methyltransferase; PMEMT_rm, Phosphatidyl-N-methylethanolamine methyltransferase; PDMEMT_rm, Phosphatidyl-N,N-dimethylethanolamine methyltransferase. inost_rm, myo-inositol [endoplasmic reticulum membrane]; pme_rm, Phosphatidyl-N-methylethanolamine; h_rm, H+; agnp_rm, Acylglycerone phosphate; nadph_rm, NADPH; ahcys_rm, S-adenosyl-L-homocysteine; pc_rm, Phosphatidylcholine; dhap_c, dihydroxyacetone phosphate [cytoplasm]; cdpdag_rm, CDP-diacylglycerol; cdpchol_rm, CDP-choline; ps_rm, Phosphatidyl-L-serine; mi1p__D_c, 1D-myo-inositol 1-phosphate; pdme_rm, Phosphatidyl-N,N-dimethylethanolamine; coa_rm, coenzyme A; pail_rm, 1-Phosphatidyl-1D-myo-inositol; pi_rm, phosphate; ser__L_rm, L-serine; inost_c, myo-inositol; nad_c, NAD; cdpea_rm, CDP-ethanolamine; h_c, H+; glyc3p_rm, glycerol 3-phosphate; acylcoa_rm, Acyl-CoA; ppi_rm, diphosphate; pe_rm, Phosphatidylethanolamine; h2o_c, H2O; g6p_c, D-glucose 6-phosphate; glyc3p_c, glycerol 3-phosphate [cytoplasm]; dhap_rm, dihydroxyacetone phosphate [endoplasmic reticulum membrane]; pa_rm, Phosphatidate; amet_rm, S-adenosyl-L-methionine; tag_rm, Triacylglycerol; nadh_c, NADH; h2o_rm, H2O; ctp_rm, CTP; nadp_rm, NADP(+); pi_c, phosphate; cmp_rm, CMP; 1agp_rm, 1-Acyl-sn-glycerol 3-phosphate; dag_rm, Diacylglycerol.

**Additional file 8. Table S2.** Media formulations of different C/N ratios used in this study (all amounts listed under C/N 5, 100 and 150 columns are volume amounts in mL to make up a total solution of 100 mL).

**Additional file 9.** **Table S3.** Composition of internal standards spiked-in during lipidomic extraction.

**Additional file 10. Table S4.** Intracellular lipidomic data of R. toruloides IFO0880 grown in nitrogen-sufficient and nitrogen-deficient media at timepoints described in Figure 1.

## Data Availability

RNA-seq data is made available at https://www.ncbi.nlm.nih.gov/bioproject/PRJNA1000066 and lipidomic data is available in the supplementary material. *R. toruloides* IFO0880 strain can be made available upon request.
