# Supplementary material for: Nitrogen starvation causes lipid remodeling in Rhodotorula toruloides
Source: Microb Cell Fact. 2024 May 17;23:141. doi: 10.1186/s12934-024-02414-0 (PMC11102182; doi:10.1186/s12934-024-02414-0)
Supplement: Supplementary file 1 — Additional file 1. Figure S1. Clustered heatmap of the RNA-seq expression data collected across different media conditions and different timepoints. The x-axis represents all the samples with individual replicates denoting the different growth media conditions and timepoints of sampling. The hierarchical dendrogram for the x-axis was computed using Euclidean distances between each pairwise combination of the samples’ mRNA expression profile. The y-axis denotes the subset of mRNA that had accompanying annotations. The hierarchical dendrogram for the y-axis was computed using the Pearson’s correlation matrix computed between pairwise combination of each mRNA (which was expressed as a numeric vector containing its expression count in each sample). The mRNA and samples were each assigned cluster identities (C1, C2, C3 and S1, S2, S3, S4, S5 respectively) as well as color labels along the left and top edges to distinguish the different clusters more easily. Further, a label of “High-N” and “Low-N” was assigned to cluster samples that were estimated to be in the nitrogen-sufficient or nitrogen-deficient regimes, respectively. From inspection of data, the mRNA belonging to cluster C1 showed a decrease in expression when switching from high nitrogen to low nitrogen media, cluster C2 showed an approximately constant expression across high and low nitrogen conditions, and cluster C3 showed an increase in expression when switching from high nitrogen to nitrogen starvation conditions. The information of mRNA identities that belong in these three clusters can be found in Additional File 2: Table S1. [file 12934_2024_2414_MOESM1_ESM.docx]

Figure S1. Clustered heatmap of the RNA-seq expression data collected across different media conditions and different timepoints. The x-axis represents all the samples with individual replicates denoting the different growth media conditions and timepoints of sampling. The hierarchical dendrogram for the x-axis was computed using Euclidean distances between each pairwise combination of the samples’ mRNA expression profile. The y-axis denotes the subset of mRNA that had accompanying annotations. The hierarchical dendrogram for the y-axis was computed using the Pearson’s correlation matrix computed between pairwise combination of each mRNA (which was expressed as a numeric vector containing its expression count in each sample). The mRNA and samples were each assigned cluster identities (C1, C2, C3 and S1, S2, S3, S4, S5 respectively) as well as color labels along the left and top edges to distinguish the different clusters more easily. Further, a label of “High-N” and “Low-N” was assigned to cluster samples that were estimated to be in the nitrogen-sufficient or nitrogen-deficient regimes, respectively. From inspection of data, the mRNA belonging to cluster C1 showed a decrease in expression when switching from high nitrogen to low nitrogen media, cluster C2 showed an approximately constant expression across high and low nitrogen conditions, and cluster C3 showed an increase in expression when switching from high nitrogen to nitrogen starvation conditions. The information of mRNA identities that belong in these three clusters can be found in Additional File 3: Table S1.
