# Supplementary material for: Nitrogen starvation causes lipid remodeling in Rhodotorula toruloides
Source: Microb Cell Fact. 2024 May 17;23:141. doi: 10.1186/s12934-024-02414-0 (PMC11102182; doi:10.1186/s12934-024-02414-0)
Supplement: Supplementary file 3 — Additional file 3. Figure S2. Gene set analysis of nitrogen limitation in R. toruloides IFO0880. The differentially expressed genes were sampled at a timepoint of A). 8 hours, and B). 12 hours, and contrasted between nutrient limited conditions of C/N 100 and 150 versus C/N 5 (which served as a baseline). Since the same set of genes were differentially expressed in both C/N 100 and 150 in comparison with C/N 5, the conditions could be plotted interchangeably [file 12934_2024_2414_MOESM3_ESM.docx]

Figure S2. Gene set analysis of nitrogen limitation in R. toruloides IFO0880. The differentially expressed genes were sampled at a timepoint of A). 8 hours, and B). 12 hours, and contrasted between nutrient limited conditions of C/N 100 and 150 versus C/N 5 (which served as a baseline). Since the same set of genes were differentially expressed in both C/N 100 and 150 in comparison with C/N 5, the conditions could be plotted interchangeably.
