# Supplementary material for: Nitrogen starvation causes lipid remodeling in Rhodotorula toruloides
Source: Microb Cell Fact. 2024 May 17;23:141. doi: 10.1186/s12934-024-02414-0 (PMC11102182; doi:10.1186/s12934-024-02414-0)
Supplement: Supplementary file 4 — Additional file 4. Figure S3. Lipidomic analysis of IFO0880 strain cultivated in different growth media containing C/N ratios of 5, 100 and 150, and sampled at various timepoints. Quantified lipid classes of all three growth conditions sampled after A) 8 hours, B) 12 hours and C) 36 hours of growth. [file 12934_2024_2414_MOESM4_ESM.docx]

Figure S3. Lipidomic analysis of IFO0880 strain cultivated in different growth media containing C/N ratios of 5, 100 and 150, and sampled at various timepoints. Quantified lipid classes of all three growth conditions sampled after A) 8 hours, B) 12 hours and C) 36 hours of growth
