# Supplementary material for: Nitrogen starvation causes lipid remodeling in Rhodotorula toruloides
Source: Microb Cell Fact. 2024 May 17;23:141. doi: 10.1186/s12934-024-02414-0 (PMC11102182; doi:10.1186/s12934-024-02414-0)
Supplement: Supplementary file 5 — Additional file 5. Figure S4. Lipidomic analysis of IFO0880 grown in C/N 100 and 150 culture conditions and sampled at a timepoint of 88 hours (oleaginous phase). [file 12934_2024_2414_MOESM5_ESM.docx]

Figure S4. Lipidomic analysis of IFO0880 grown in C/N 100 and 150 culture conditions and sampled at a timepoint of 88 hours (oleaginous phase).
