# Supplementary material for: Nitrogen starvation causes lipid remodeling in Rhodotorula toruloides
Source: Microb Cell Fact. 2024 May 17;23:141. doi: 10.1186/s12934-024-02414-0 (PMC11102182; doi:10.1186/s12934-024-02414-0)
Supplement: Supplementary file 6 — Additional file 6. File S5. Overlap of cluster identities of mRNA. The x-axis displays the 3 major mRNA clusters in the clustermap integrating multiomics data. The y-axis displays the distribution of these mRNA into the 3 major mRNA clusters from the RNA-seq clustering analysis. [file 12934_2024_2414_MOESM6_ESM.docx]

Figure S5. Overlap of cluster identities of mRNA. The x-axis displays the 3 major mRNA clusters in the clustermap integrating multiomics data. The y-axis displays the distribution of these mRNA into the 3 major mRNA clusters from the RNA-seq clustering analysis.
