# Supplementary material for: Nitrogen starvation causes lipid remodeling in Rhodotorula toruloides
Source: Microb Cell Fact. 2024 May 17;23:141. doi: 10.1186/s12934-024-02414-0 (PMC11102182; doi:10.1186/s12934-024-02414-0)
Supplement: Supplementary file 7 — Additional file 7. File S6. The reactions and metabolites (lipids) found in each of these quadrants were then assigned a color and these colors were superimposed onto a pathway map depicting the lipid biosynthetic pathways, extracted from a genome-scale model of R. toruloides (Dinh et al). The pathway visualization shown here is for C/N of 150. The Escher maps for visualization were adapted from the genome-scale model developed by Dinh et al. Reactions displayed using green are correlated positively with storage lipid increase (DAGs and TAGs), whereas those displayed with red are negatively correlated with storage lipid accumulation. The linear chain of reactions upstream of storage lipid synthesis were mostly positively correlated as expected. The reactions of phospholipid synthesis that utilize the same precursors as storage lipids, thus withdrawing flux from storage lipids synthesis are negatively correlated with storage lipid increase. This observation was verified from the lipidomic data where phospholipid values dropped in the oleaginous phase where DAG and TAG increased. A pattern of selective regulation of reactions within lipid biosynthetic pathways in the context of storage lipid accumulation was observed. The phospholipid biosynthetic pathways were negatively correlated to storage lipid accumulation in both maps (reaction pathway on the right), whereas reactions upstream of the storage lipid synthesis for acylation of the glycerol backbone were upregulated in both (bottom left). These patterns supported the observation that lipid accumulation during nitrogen starvation was accompanied by a rerouting of carbon flux towards storage lipids and away from glycerophospholipids. G3PD1i_c, glycerol-3-phosphate dehydrogenase (NAD); DHAPt_c_rm, dihydroxyacetone phosphate transport; DHAPAT_rm, Dihydroxyacetone phosphate acyltransferase; AGNPR_rm, Acylglycerone-phosphate reductase; GLYC3Pt_c_rm, glycerol 3-phosphate transport; G3PAT_rm, Glycerol-3-phosphate acyltrans [file 12934_2024_2414_MOESM7_ESM.docx]

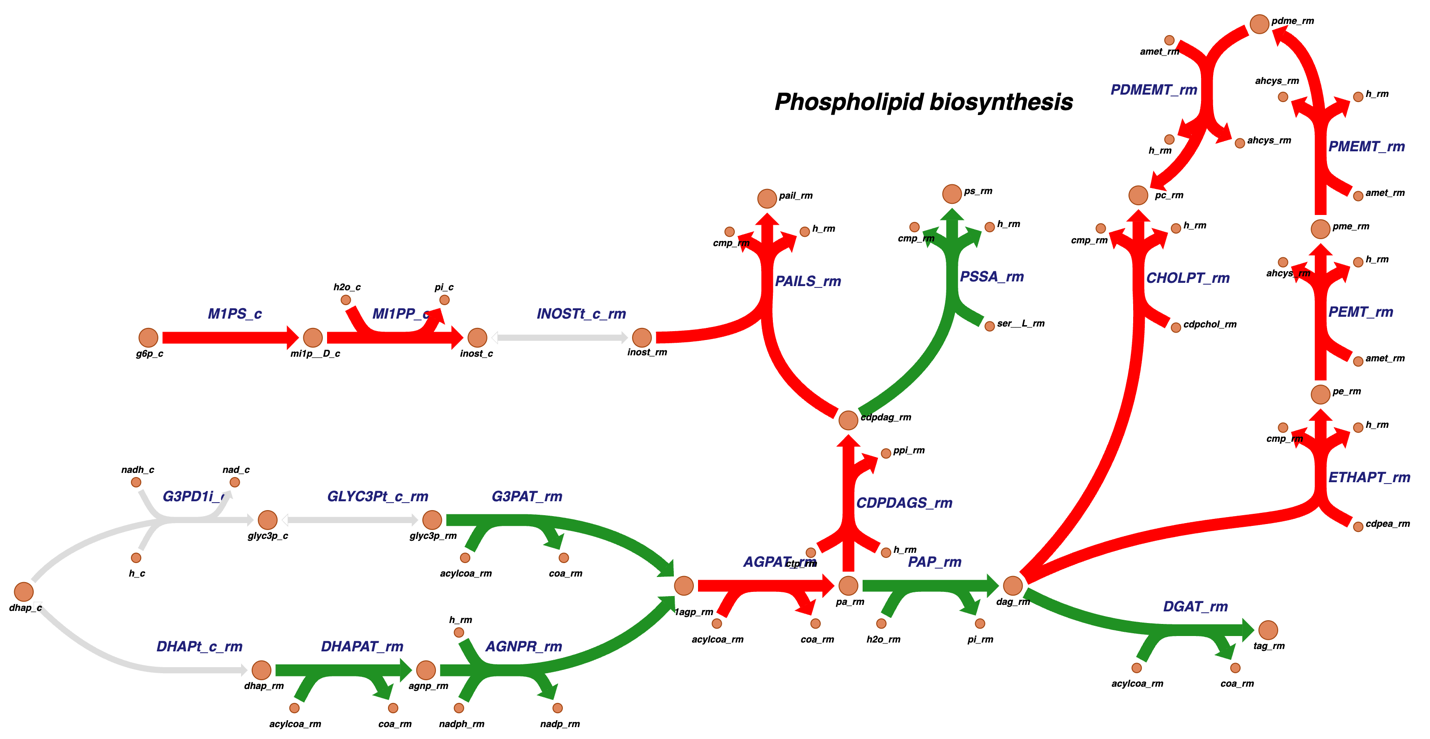


Figure S6. The reactions and metabolites (lipids) found in each of these quadrants were then assigned a color and these colors were superimposed onto a pathway map depicting the lipid biosynthetic pathways, extracted from a genome-scale model of *R. toruloides* (Dinh et al). The pathway visualization shown here is for C/N of 150. The Escher maps for visualization were adapted from the genome-scale model developed by Dinh et al. Reactions displayed using green are correlated positively with storage lipid increase (DAGs and TAGs), whereas those displayed with red are negatively correlated with storage lipid accumulation. The linear chain of reactions upstream of storage lipid synthesis were mostly positively correlated as expected. The reactions of phospholipid synthesis that utilize the same precursors as storage lipids, thus withdrawing flux from storage lipids synthesis are negatively correlated with storage lipid increase. This observation was verified from the lipidomic data where phospholipid values dropped in the oleaginous phase where DAG and TAG increased. A pattern of selective regulation of reactions within lipid biosynthetic pathways in the context of storage lipid accumulation was observed. The phospholipid biosynthetic pathways were negatively correlated to storage lipid accumulation in both maps (reaction pathway on the right), whereas reactions upstream of the storage lipid synthesis for acylation of the glycerol backbone were upregulated in both (bottom left). These patterns supported the observation that lipid accumulation during nitrogen starvation was accompanied by a rerouting of carbon flux towards storage lipids and away from glycerophospholipids. G3PD1i_c, glycerol-3-phosphate dehydrogenase (NAD); DHAPt_c_rm, dihydroxyacetone phosphate transport; DHAPAT_rm, Dihydroxyacetone phosphate acyltransferase; AGNPR_rm, Acylglycerone-phosphate reductase; GLYC3Pt_c_rm, glycerol 3-phosphate transport; G3PAT_rm, Glycerol-3-phosphate acyltransferase; AGPAT_rm, 1-Acyl-sn-glycerol-3-phosphate acyltransferase; PAP_rm, PA phosphatase; DGAT_rm, Diacylglycerol acyltransferase; CDPDAGS_rm, CDP-diacylglycerol synthase; PSSA_rm, PS synthase; PAILS_rm, PI synthase; INOSTt_c_rm, myo-inositol transport; MI1PP_c, myo-inositol 1-phosphatase; M1PS_c, myo-inositol-1-phosphate synthase; CHOLPT_rm, Cholinephosphotransferase; ETHAPT_rm, Ethanolaminephosphotransferase; PEMT_rm, PE methyltransferase; PMEMT_rm, Phosphatidyl-N-methylethanolamine methyltransferase; PDMEMT_rm, Phosphatidyl-N,N-dimethylethanolamine methyltransferase. inost_rm, myo-inositol [endoplasmic reticulum membrane]; pme_rm, Phosphatidyl-N-methylethanolamine; h_rm, H+; agnp_rm, Acylglycerone phosphate; nadph_rm, NADPH; ahcys_rm, S-adenosyl-L-homocysteine; pc_rm, Phosphatidylcholine; dhap_c, dihydroxyacetone phosphate [cytoplasm]; cdpdag_rm, CDP-diacylglycerol; cdpchol_rm, CDP-choline; ps_rm, Phosphatidyl-L-serine; mi1p__D_c, 1D-myo-inositol 1-phosphate; pdme_rm, Phosphatidyl-N,N-dimethylethanolamine; coa_rm, coenzyme A; pail_rm, 1-Phosphatidyl-1D-myo-inositol; pi_rm, phosphate; ser__L_rm, L-serine; inost_c, myo-inositol; nad_c, NAD; cdpea_rm, CDP-ethanolamine; h_c, H+; glyc3p_rm, glycerol 3-phosphate; acylcoa_rm, Acyl-CoA; ppi_rm, diphosphate; pe_rm, Phosphatidylethanolamine; h2o_c, H2O; g6p_c, D-glucose 6-phosphate; glyc3p_c, glycerol 3-phosphate [cytoplasm]; dhap_rm, dihydroxyacetone phosphate [endoplasmic reticulum membrane]; pa_rm, Phosphatidate; amet_rm, S-adenosyl-L-methionine; tag_rm, Triacylglycerol; nadh_c, NADH; h2o_rm, H2O; ctp_rm, CTP; nadp_rm, NADP(+); pi_c, phosphate; cmp_rm, CMP; 1agp_rm, 1-Acyl-sn-glycerol 3-phosphate; dag_rm, Diacylglycerol.
