# Supplementary material for: Nitrogen starvation causes lipid remodeling in Rhodotorula toruloides
Source: Microb Cell Fact. 2024 May 17;23:141. doi: 10.1186/s12934-024-02414-0 (PMC11102182; doi:10.1186/s12934-024-02414-0)
Supplement: Supplementary file 8 — Additional file 8. Table S2. Media formulations of different C/N ratios used in this study (all amounts listed under C/N 5, 100 and 150 columns are volume amounts in mL to make up a total solution of 100 mL). [file 12934_2024_2414_MOESM8_ESM.docx]

Table S1. Media formulations of different C/N ratios used in this study (all amounts listed under C/N 5, 100 and 150 columns are volume amounts in mL to make up a total solution of 100 mL).

| **Stocks** | **Final concentration** | **C/N 5** | **C/N 100** | **C/N 150** |
| --- | --- | --- | --- | --- |
| Glucose (200 g/L) | 20 g/L | 10 | 10 | 10 |
| YNBwaaas (17 g/L) | 1.7 g/L | 10 | 10 | 10 |
| NH_4_Cl (61 g/L) | 6.1 / 0.305 / 0.203 g/L | 10 | 0.5 | 0.33 |
| Na_2_HPO_4_ (0.5M) | 25 mM | 5 | 5 | 5 |
| KH_2_PO_4_ (1M) | 125 mM (pH = 5.6) | 12.5 | 12.5 | 12.5 |
| Water |  | 52.5 | 62 | 62.17 |

*YNBwaaas: Yeast Nitrogen Base without amino acids or ammonium sulphate
