# Supplementary material for: Nitrogen starvation causes lipid remodeling in Rhodotorula toruloides
Source: Microb Cell Fact. 2024 May 17;23:141. doi: 10.1186/s12934-024-02414-0 (PMC11102182; doi:10.1186/s12934-024-02414-0)
Supplement: Supplementary file 9 — Additional file 9. Table S3. Composition of internal standards spiked-in during lipidomic extraction. [file 12934_2024_2414_MOESM9_ESM.docx]

Table S2. Composition of internal standards spiked-in during lipidomic extraction.

| Class | Component | Amount (pmol) |
| --- | --- | --- |
| PC | IS PC 15:0/18:1-d7 | 200.10 |
| PE | IS PE 17:0/14:1 | 14.58 |
| PS | IS PS 17:0/14:1 | 13.44 |
| PG | IS PG 15:0/18:1-d7 | 34.96 |
| PI | IS PI 17:0/14:1 | 12.59 |
| PA | IS PA 15:0/18:1-d7 | 10 |
| LPC | IS LPC 18:1-d7 | 45 |
| LPE | IS LPE 18:1-d7 | 10.08 |
| EE | IS CE 18:1-d7 | 500.40 |
| DAG | IS DAG 15:0/18:1-d7 | 14.98 |
| TAG | IS TAG 15:0/18:1-d7/15:0 | 65.04 |
| Cer | IS Cer 18:1;2/17:0 | 226.48 |
| IPC | IS PI 17:0/14:1 | 12.59 |
| MIPC | IS PI 17:0/14:1 | 12.59 |
| MIP2C | IS PI 17:0/14:1 | 12.59 |
| Ergosterol | IS Cholesterol-d7 | 250.12 |
| LPA | IS PA 15:0/18:1-d7 | 10 |
| LPI | IS PI 17:0/14:1 | 12.59 |
| LPS | IS PS 17:0/14:1 | 20.16 |
